# Shaping corticospinal pathways in virtual reality: effects of task complexity and sensory feedback during mirror therapy in neurologically intact individuals

**DOI:** 10.1186/s12984-024-01454-2

**Published:** 2024-09-04

**Authors:** Trevor A. Norris, Thomas E. Augenstein, Kazandra M. Rodriguez, Edward S. Claflin, Chandramouli Krishnan

**Affiliations:** 1 Neuromuscular & Rehabilitation Robotics Laboratory (NeuRRo Lab), Michigan Medicine, University of Michigan, 325 E Eisenhower Parkway (Room 3013), Ann Arbor, MI 48108 USA; 2 Robotics Department, University of Michigan, Ann Arbor, MI USA; 3 Department of Mechanical Engineering, University of Michigan, Ann Arbor, MI USA; 4 School of Kinesiology, University of Michigan, Ann Arbor, MI USA; 5 Department of Biomedical Engineering, University of Michigan, Ann Arbor, MI USA; 6 Department of Physical Therapy, University of Michigan-Flint, Flint, MI USA; 7 Physical Medicine and Rehabilitation, Michigan Medicine, Ann Arbor, MI USA

## Abstract

**Background:**

Restoration of limb function for individuals with unilateral weakness typically requires volitional muscle control, which is often not present for individuals with severe impairment. Mirror therapy—interventions using a mirror box to reflect the less-impaired limb onto the more-impaired limb—can facilitate corticospinal excitability, leading to enhanced recovery in severely impaired clinical populations. However, the mirror box applies limitations on mirror therapy, namely that all movements appear bilateral and are confined to a small area, impeding integration of complex activities and multisensory feedback (e.g., visuo-tactile stimulation). These limitations can be addressed with virtual reality, but the resulting effect on corticospinal excitability is unclear.

**Objective:**

Examine how virtual reality-based unilateral mirroring, complex activities during mirroring, and visuo-tactile stimulation prior to mirroring affect corticospinal excitability.

**Materials and methods:**

Participants with no known neurological conditions (*n* = 17) donned a virtual reality system (NeuRRoVR) that displayed a first-person perspective of a virtual avatar that matched their motions. Transcranial magnetic stimulation-induced motor evoked potentials in the nondominant hand muscles were used to evaluate corticospinal excitability in four conditions: resting, mirroring, mirroring with prior visuo-tactile stimulation (mirroring + TACT), and control. During mirroring, the movements of each participant’s dominant limb were reflected onto the nondominant limb of the virtual avatar, and the avatar’s dominant limb was kept immobile (i.e., unilateral mirroring). The mirroring + TACT condition was the same as the mirroring condition, except that mirroring was preceded by visuo-tactile stimulation of the nondominant limb. During the control condition, unilateral mirroring was disabled. During all conditions, participants performed simple (flex/extend fingers) and complex (stack virtual blocks) activities.

**Results:**

We found that unilateral mirroring increased corticospinal excitability compared to no mirroring (*p* < 0.001), complex activities increased excitability compared to simple activities during mirroring (*p* < 0.001), and visuo-tactile stimulation prior to mirroring decreased excitability (*p* = 0.032). We also found that these features did not interact with each other.

**Discussions:**

The findings of this study shed light onto the neurological mechanisms of mirror therapy and demonstrate the unique ways in which virtual reality can augment mirror therapy. The findings have important implications for rehabilitation for design of virtual reality systems for clinical populations.

## Background

Individuals who suffer from a neurological injury, such as stroke or cerebral palsy, often experience unilateral weakness (hemiparesis) [1–3]. For instance, 65% of stroke survivors are unable to fully incorporating their affected limb into activities of daily living six months after the event [4]. This impaired motor control often results in increased reliance of the less-impaired limb, inhibiting restoration of normal movement of the more-impaired limb [5, 6]. Common methods of rehabilitation utilize task-oriented and/or strength training [7–9], but these interventions frequently require some amount of initial voluntary movement, which is often not present in individuals with severe impairment [10]. Because of this, it is pertinent that researchers investigate new methods of restoring motor function in individuals with severe hemiparesis.

Mirror therapy has been previously presented as a useful intervention for patients with severe hemiparesis [11–13]. Conventionally, mirror therapy uses a mirror box to block the patient’s view of their more-impaired limb and reflect an image of the less-impaired limb across the midline. As a result, the patient sees their less-impaired limb (i.e., mirrored limb) and a virtual representation of their more-impaired limb (i.e., the mirroring limb) moving synchronously. Previous studies have shown that mirror therapy increases excitability of the corticospinal tract contralateral to the mirroring limb [14, 15], enhances functional connectivity between the contralateral sensorimotor cortex and the supplementary motor area [16], and is a useful tool for functional recovery in clinical populations [12].

The effectiveness of mirror therapy is believed to rely on the patient’s perception that they control the mirroring limb (i.e., agency) and it is part of their body (i.e., ownership) [17], but these perceptions may be limited by the mirror box itself. First, the mirror box causes all movements to appear as bilateral instead of unilateral movements of the mirroring limb. This is because the patient’s perception of the mirrored limb is unaffected by the illusion (i.e., the less impaired limb is usually visible through their direct or peripheral vision). Visual feedback of the mirrored limb may reduce the user’s focus on the mirroring limb and increase their awareness that the mirroring limb is an illusion [18]. Second, limitations in the size and location of the mirror impedes isolated integration of the mirroring limb into complex, task-relevant activities such as multi-joint movements and/or object manipulation. Complex tasks can potentially augment mirror therapy, as they can both heighten the patient’s focus and increase the salience of training, which are both critical for facilitating neural plasticity [19–21]. Additionally, complex activities with the unimpaired limb have been previously shown to lead to gains in the impaired limb through a phenomenon known as “cross-education” [22], thus, potentially supplementing mirror therapy. Last, the mirror limits the ready incorporation of multisensory feedback (e.g., visuo-tactile stimulation) into the illusion. Previous studies in mirror therapy have shown that visuo-tactile feedback can increase limb ownership [23]. However, producing a convincing multisensory feedback illusion with a physical mirror requires the use of a prosthetic limb that is receiving the visuo-tactile stimulation at the same time as the physical limb [24]. This prosthetic limb must be a similar size [24] and color [25] of the patient’s physical limb and must have a similar location [26] and orientation [27] to the physical limb [24]. This requires clinics to have several realistic prosthetic limbs of differing size and color and requires clinicians to invest the necessary time to carefully set up the illusion, which could be quite difficult in a health care system affected by resource and time constraints [28].

Virtual reality (VR) systems—systems that display a fully virtual and interactive environment to the user—have unique capabilities that may address some of these shortcomings in conventional mirror therapy. For instance, these systems often include fully immersive headsets that can alter the movements of both the mirrored and mirroring limbs to generate unilateral movements of the mirroring limb. Furthermore, interactive VR environments can more easily enable complex activities into mirroring because the illusion is not limited by a physical mirror. Finally, many VR systems offer external tracking capabilities that can locate physical objects relative to the headset, thus, enabling the mirroring and more-impaired limb to synchronously interact with these objects for multisensory feedback (i.e., visuo-tactile stimulation). While these features represent exciting possibilities for enhancing mirror therapy, it is unclear whether they modulate corticospinal excitability i.e., the excitability of the neural connection between the motor cortex and the contralateral limb (i.e., the corticospinal tract). Corticospinal excitability is often measured by transcranial magnetic stimulation (TMS), where a magnetic pulse over the primary motor cortex causes efferent volleys (i.e., motor evoked potentials) in the muscles of the contralateral limb. Previous studies have reported that corticospinal excitability is reduced in the affected primary motor cortex following neurological injuries such as stroke [29]. Corticospinal excitability is also a commonly used indicator for neuroplasticity and motor recovery in individuals with neurological injury, as the presence of MEPs and higher MEP amplitudes are predictive of better functional outcomes [30–32]. Thus, investigating the effects of VR during mirror therapy on corticospinal excitability could lend insight on its potential to restore function following neurological injury.

Therefore, the objective of the current study was to examine if VR-enabled alterations to mirror therapy alter corticospinal excitability. Here, participants interacted with a VR system capable of producing a unilateral mirror illusion while TMS was used to stimulate the primary motor cortex contralateral to the mirroring limb. During the experiment, participants performed both complex, task-oriented activities and simple, task-irrelevant activities and we modulated the mirror illusion and multisensory stimulation prior to mirroring. We structured the study to examine how [1] unilateral mirroring, [2] complex tasks during mirroring, and [3] multisensory feedback prior to mirroring influenced corticospinal excitability. We found that the unilateral mirror illusion increased corticospinal excitability relative to no mirroring illusion. Interestingly, we found that complex, task-oriented activities during mirroring increased corticospinal excitability relative to simple movements, and multisensory feedback prior to mirroring decreased corticospinal excitability as compared to the mirroring alone, although these features generally did not interact with each other. These findings establish the unique capabilities of virtual reality to alter mirror therapy.

## Materials and methods

### Participants

Seventeen adults with no known neurological condition were recruited to participant in this study (age: 20.6 ± 2.4 yrs, 10 male, 7 female, 14 right-hand dominant, determined by self-reported preference of which hand is used to throw a ball [33, 34]). This sample size provided a power (1- *β*) > 90% to detect statistical significance at *α* ≤ 0.05 between mirroring and no mirroring conditions based on data from four pilot participants using paired *t*-tests (effect size ‘dz’ = 0.848, which was 50% of the effect size observed in the preliminary data) in G*Power (Version 3.1.9.6). Potential participants were excluded from the study if they were unable to think clearly or remember (Mini-mental state examination < 22) [35], had any major medical condition that would significantly affect the results of the study, or had one of the following TMS-related contraindications: (1) pregnant or actively trying to conceive, (2) unexplained recurrent headaches, (3) recent (< 6 months) history of seizure, (4) a history or skull fracture or head injury, (5) have metal implants in the skull, or (6) have a cardiac pacemaker. All participants reviewed and signed a written informed consent document approved by the University of Michigan Institutional Review Board (IRBMED).

### Experimental set-up and protocol

**Fig. 1:**
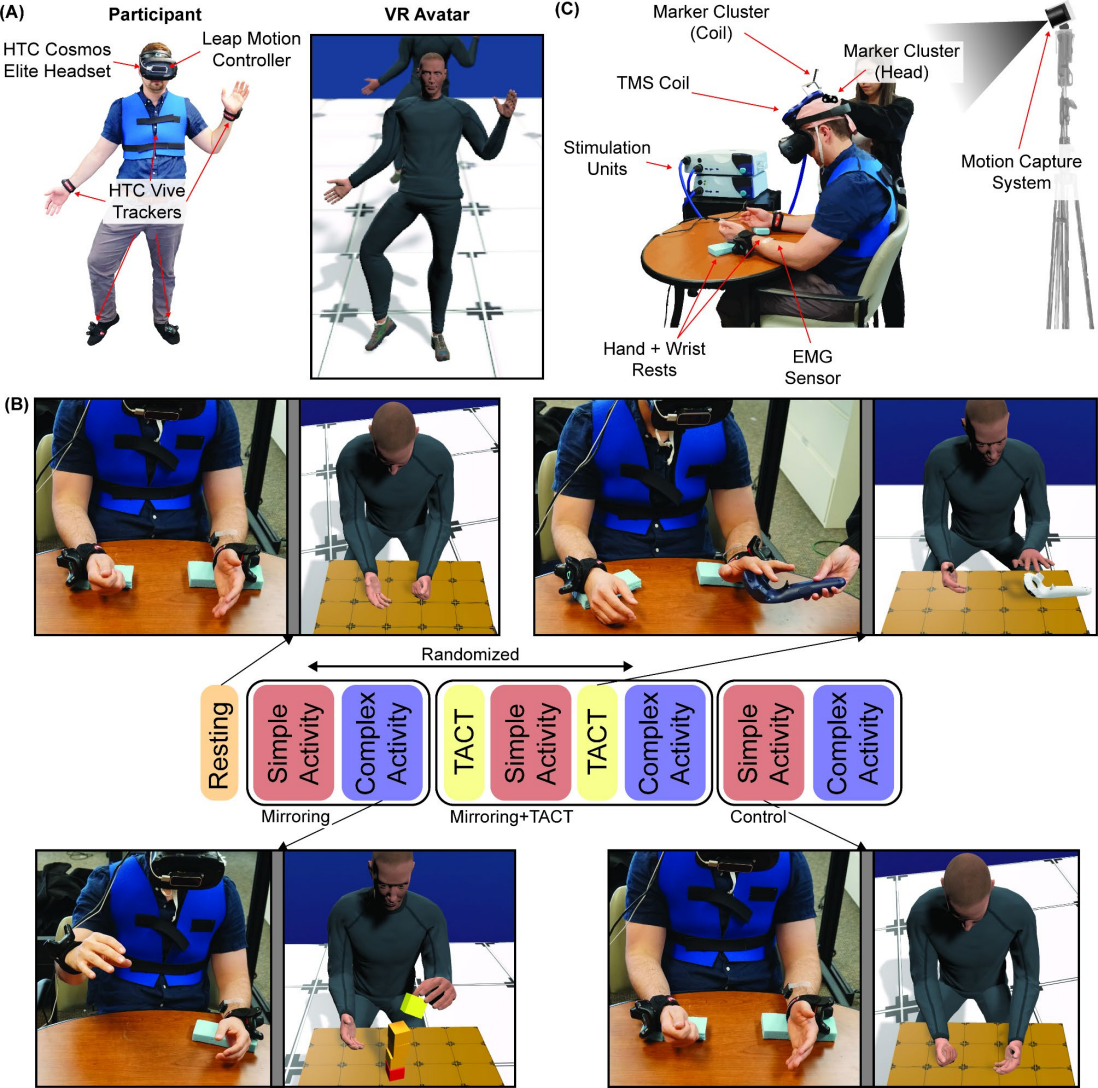
(**A**) Demonstration of NeuRRoVR, a virtual reality platform that can display and mirror the trunk, upper extremity, lower extremity, and finger movements. (**B**) Experimental protocol. Participants used NeuRRoVR in four conditions: rest, mirroring, mirroring + TACT, and control. During mirroring and mirroring + TACT, movements of the participant’s dominant limb were reflected onto the avatar’s nondominant limb and the avatar’s dominant limb was immobilized (i.e., unilateral mirroring). Prior to the mirroring + TACT condition, participants experienced a two-minute period of visuo-tactile stimulation. In the control condition, unilateral mirroring was disabled. During the mirroring, mirroring + TACT, and control conditions, participants performed a simple activity (finger flexion/extension) and a complex, task-oriented activity (stacking virtual blocks). (**C**) Transcranial magnetic stimulation experimental set-up. The TMS coil was positioned over the primary motor cortex contralateral to the nondominant (mirroring) limb, and motor evoked potentials were measured with a surface electromyography sensor on the flexor digitorum superficialis. A motion capture system measured the position and orientation of the coil and the participant’s head for consistent coil placement throughout the experiment

We used our custom virtual reality program, NeuRRoVR, to create a virtual environment for the experiment and a virtual representation of the participant within the environment (i.e., their avatar) that tracked their movements (Fig. 1A). The development, operating principles, and validation of NeuRRoVR has been discussed in prior work [36], but to summarize here, NeuRRoVR is an immersive virtual reality (VR) system designed for performing mirror therapy. NeuRRoVR consists of an immersive virtual reality headset (HTC Vive Cosmos Elite, HTC, Taoyuan City, Taiwan), five wearable sensors (HTC Vive Tracker 2.0, HTC, Taoyuan City, Taiwan), an infrared camera-based motion-tracker (Leap Motion Controller, Ultraleap, San Francisco, CA) mounted to the anterior surface of the headset, and a desktop computer. The desktop computer interfaces with these components to create the virtual environment and avatar using Unity game engine (Unity Technologies, Version 2018.2.12f1). The headset was placed on the participant’s head to track the participant’s head orientation and display an immersive, first-person perspective of the virtual environment and avatar to the participant. One HTC Vive Tracker was attached to the participant’s trunk to track the participant’s trunk position and orientation. The remaining four HTC Vive trackers were attached to the participant’s wrist and feet to track their forearms and feet, respectively. Following the input of the participant’s height and all inter-joint distances, NeuRRoVR used these five trackers and inverse kinematics to track the trunk movements, shoulder and elbow movements in both upper extremities, and hip, knee, and ankle movements in both lower extremities. NeuRRoVR used the infrared camera-based motion tracker (i.e., Leap Motion Controller) to track the participant’s wrists, hands, and fingers. During the experiment, a humanoid avatar was used whose skin tone was set to match the skin tone of the participant to increase the participant’s sense of ownership of the avatar’s limbs [25]. The accuracy of the systems kinematics relative to the user’s joint motion has been established in our previous work [36].

The experiment consisted of four conditions: a resting condition, a mirroring condition, a mirroring with visuo-tactile stimulation (mirroring + TACT) condition, and a control condition (Fig. 1(B)). During the resting condition, participants sat with their shoulders in neutral position, elbows flexed to 90°, forearms slightly supinated, hands open and relaxed, and wrists and hands resting on foam pads placed on the table. In NeuRRoVR, a virtual table of a similar color was displayed in front of the participant whose position and height approximately matched the physical table. During this condition, participants were instructed to relax their arms and look at their nondominant arm, which was set in NeuRRoNav to exactly match the participants movements. In the mirroring condition, unilateral mirroring was enabled in NeuRRoVR so that the movements of the avatar’s nondominant (mirroring) limb matched the movements of the participant’s dominant (mirrored) limb; the avatar’s dominant limb remained immobile in the resting posture. During the mirroring condition, participants performed two activities: simple, task-irrelevant (i.e., not oriented to a functional task) and complex, task-relevant (i.e., oriented to a functional task). During the simple activity, participants flexed and extended the fingers of their dominant hand to the beat of an auditory metronome (60 bpm) while maintaining the resting posture with their nondominant limb. During the complex activity, two virtual blocks appeared on the virtual table in front of the avatar, and when participants stacked one block on top of the other, a new block appeared. Participants were instructed to control the avatar’s nondominant (mirroring) limb to create the tallest stack of blocks that they could in 5 min. The mirroring + TACT condition was identical to the mirroring condition except that, prior to each activity, visuo-tactile stimulation was performed on the participant’s nondominant limb. Specifically, a physical wand that appeared in the virtual environment was used to stroke the dorsal and palmar surfaces of the hand and fingers on the nondominant limb for two minutes (i.e., the participants saw the wand stroking their virtual hand and felt it stroking their physical hand). Participants were instructed to watch the avatar’s nondominant hand during this time, which appeared to be synchronously stroked. Following this, unilateral mirroring was re-enabled and each activity was conducted as previously described. The control condition was identical to the mirroring condition, except mirroring was disabled for both activities so that the participant saw the movements of their dominant limb appear as movements of the avatar’s dominant limb. The order of the mirroring and mirroring + TACT conditions were randomized to minimize the potential effects of ordering (Fig. 1(B)).

### Electromyography and transcranial magnetic stimulation

We used transcranial magnetic stimulation (TMS) to examine each participant’s corticospinal excitability during each of the conditions listed above (Fig. 1(C)). Prior to the experiment, a linen cap was secured to the participant’s skull to assist in coil positioning. The TMS pulses were delivered over the primary motor cortex contralateral to the participant’s nondominant limb using a monophasic magnetic stimulator (Magstim 200^2^, Magstim Company Ltd, Whitland, UK) and a 70 mm figure-of-eight coil. The coil was oriented such that the handle was rotated 45° from the participant’s midline and pointing posteriorly to produce posterior-to-anterior current flow in the cortex. To identify the optimal coil position (i.e., the “hot spot”), the coil was initially positioned 3 cm anterior and lateral of the participant’s vertex and then moved in small increments until the position that produced the largest MEP amplitude was found. The resting motor threshold was then established by identifying the minimum TMS stimulus intensity needed to produce a distinguishable MEP in ≥ 50% of stimulations [37–40]. This process was assisted by an adaptive threshold-hunting technique based on based on maximum-likelihood parameter estimation by sequential testing (TMS Motor Threshold Assessment Tool, MTAT 2.0, https://www.clinicalresearcher.org/software.htm) [41] while the participant sat in the resting posture. After establishing the resting motor threshold and hot spot, the stimulator intensity was increased to 120% of the threshold value, and this intensity was used for the rest of the experiment. To ensure consistent coil placement throughout the experiment, a cluster of three retroreflective markers (9 mm diameter) attached to the coil and a cluster of five retroreflective markers attached to the back of the head were tracked using an OptiTrack V120: TRIO camera and Motive Motion Capture Software (Version 1.8.0, 120 Hz). A custom-developed software for TMS navigation (NeuRRoNav) provided feedback on the position and orientation of the coil relative to hot spot [42]. Twenty stimulations were delivered during the resting condition and during each activity in the mirroring, mirroring + TACT, and control conditions. During the simple activities, stimulations were manually provided by the experimenter during the finger flexion movements. During the complex activities, stimulations were manually provided by the experimenter while the participant was either attempting to grasp the block, moving a block to the top of the stack, or placing the block on the top of the stack.

During the experiment, we recorded the motor evoked potentials (MEPs) in response to TMS using surface electromyography (EMG) from each participant’s flexor digitorum superficialis on their nondominant limb. Prior to sensor placement, the skin over each muscle belly was cleaned and prepped with alcohol pads. Following this, the wireless electrode (Trigno Avanti, Delsys, Inc., Natick, MA) was secured to the skin using self-adhesive tapes and medical tapes (Transpore Medical Tape, 3 M, Minneapolis, MI). Cotton elastic bandages were wrapped around the participants nondominant forearm to ensure good contact between the electrode and the participant’s skin, and wide tapes (Cover Roll Stretch 4”, BSN Medical, Hamburg, Germany) were used on along the edges of the bandages to ensure that the bands did not unravel during the experiment. Analog signals were low-pass filtered (500 Hz) using a National Instruments Analog Butterworth Filter (NI SCXI-1143) and then sampled at 2000 Hz using a 16-bit National Instruments Data Acquisition system (NI USB-6255). Prior to data collection, the EMG signal was visually inspected to ensure correct placement and signal quality. It is important to note that, in all conditions/activities, the participant only used their dominant limb. Therefore, their nondominant limb was always relaxed (verified via continuous visual inspection), preventing MEPs from being confounded by background muscle activity.

### Data analysis

Custom-written LabView programs were used to perform all data collection and analysis. The recorded EMG data were filtered using a zero-lag, 4th order high-pass filter with a 0.25 Hz cutoff frequency to remove DC gain. The filtered EMG data were segmented into 300 ms windows following each the stimulation and ensemble averaged to establish an average MEP for each condition/activity. The peak-to-peak MEP amplitude of the ensemble averaged MEP waveform for each condition/activity was normalized to the peak-to-peak MEP amplitude during the resting condition.

### Statistical analysis

All statistical analyses were performed using IBM SPSS Statistics (Statistical Product and Service Solution, Version 27, Chicago, IL). Descriptive statistics were computed for the MEP responses obtained during each condition/activity to evaluate the distribution and variation of the outcome variable. We performed two linear mixed-model analyses that examined (1) task complexity and mirroring and (2) visuo-tactile stimulation and task complexity during mirroring. Note that we did not perform a three-way analysis of variance as our design was not balanced with control conditions that evaluated the effects of visuo-tactile stimulation without mirroring to perform this analysis. Prior to these analyses, MEP values from surface EMG data were log-transformed to minimize skewness and heteroscedasticity [43]. To examine task complexity and mirroring, we performed a linear mixed-model analysis with task complexity (two levels, simple and complex) and mirroring (two levels, Mirroring and Control) as fixed factors and subject (i.e., participant) as a random factor. Similarly, to examine visuo-tactile stimulation and task complexity, we performed a linear mixed-model analysis with visuo-tactile stimulation (two levels, mirroring and mirroring + TACT) and task complexity (two levels, simple and complex) as fixed factors and subject as a random factor. Any significant main or interaction effects were followed by appropriate post-hoc analyses with Šidák correction. A significance level of *α* = 0.05 was used for all analyses.

## Results

**Fig. 2:**
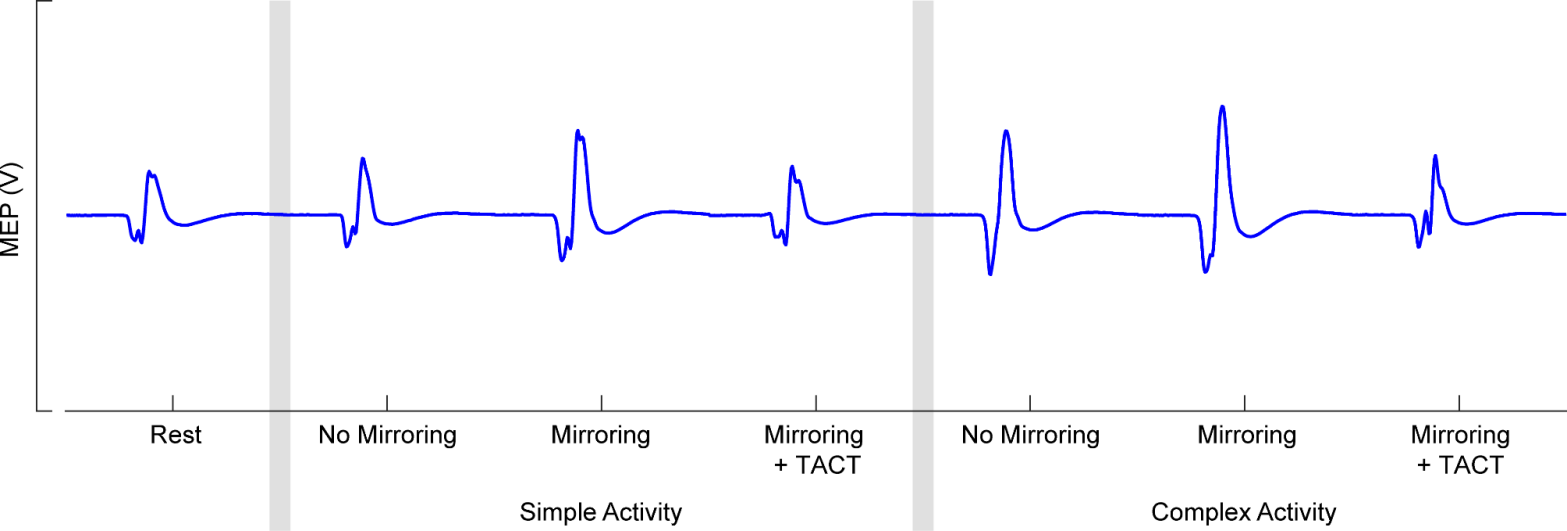
Motor evoked potentials (MEPs) from a representative participant in all conditions and activities

**Fig. 3:**
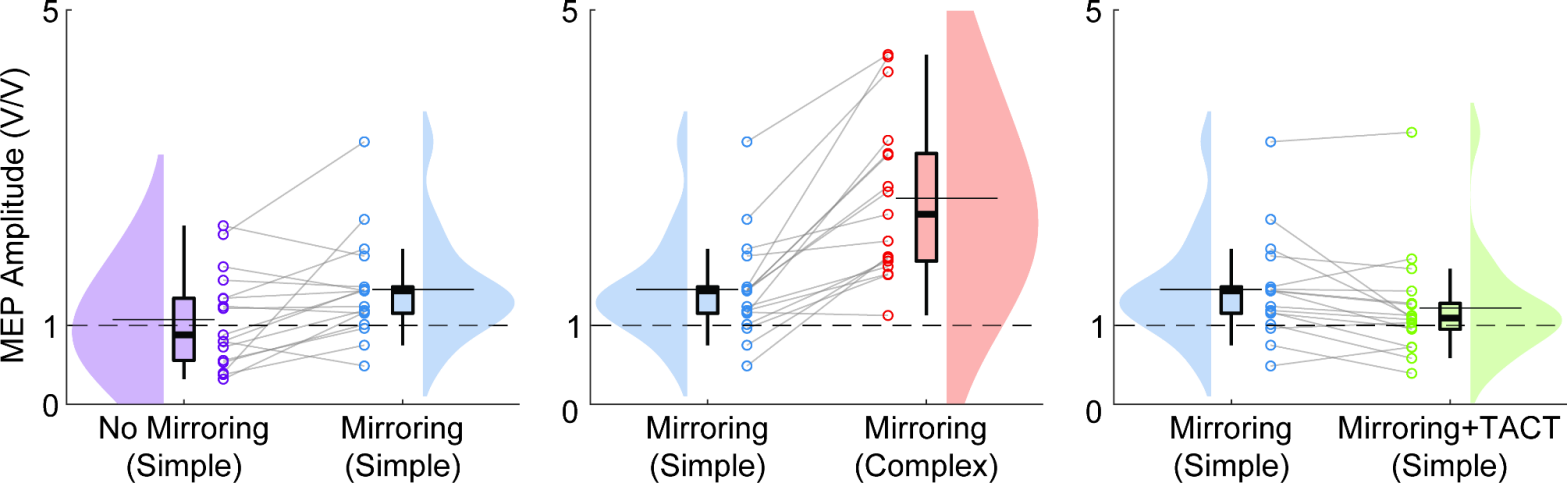
Raincloud plots [76] comparing corticospinal excitability during (Left) no mirroring during the simple activity to mirroring during the simple activity, (Middle) mirroring during the simple activity to mirroring during the complex activity, and (Right) mirroring during the simple activity to mirroring during the simple activity following visuo-tactile stimulation (TACT). Here, the thick horizontal line contained by the box-and-whisker indicates the median and the thin horizontal line extending beyond the box-and-whisker indicates the mean

Motor evoked potentials (MEPs) from a representative participant are shown in Fig. 2. Resting motor threshold for participants were 60 ± 9% of maximum stimulator output. During the control condition, the participants’ average normalized peak-to-peak MEP amplitudes were 1.07 ± 0.15 V/V (mean ± standard error of the mean) and 2.05 ± 0.23 V/V during the simple and complex activities, respectively. During the mirroring condition, the participants average normalized peak-to-peak MEP amplitudes were 1.46 ± 0.16 V/V and 2.61 ± 0.25 V/V during the simple and complex activities, respectively. During the mirroring + TACT condition, the participants average peak-to-peak MEP amplitudes were 1.22 ± 0.17 V/V and 2.45 ± 0.31 V/V during the simple and complex activities, respectively. Due to technical issues, the complex activity during the mirroring + TACT condition was not collected for one participant. A descriptive comparison of the MEPs from the different conditions/activities is shown in Fig. 3.

**Fig. 4:**
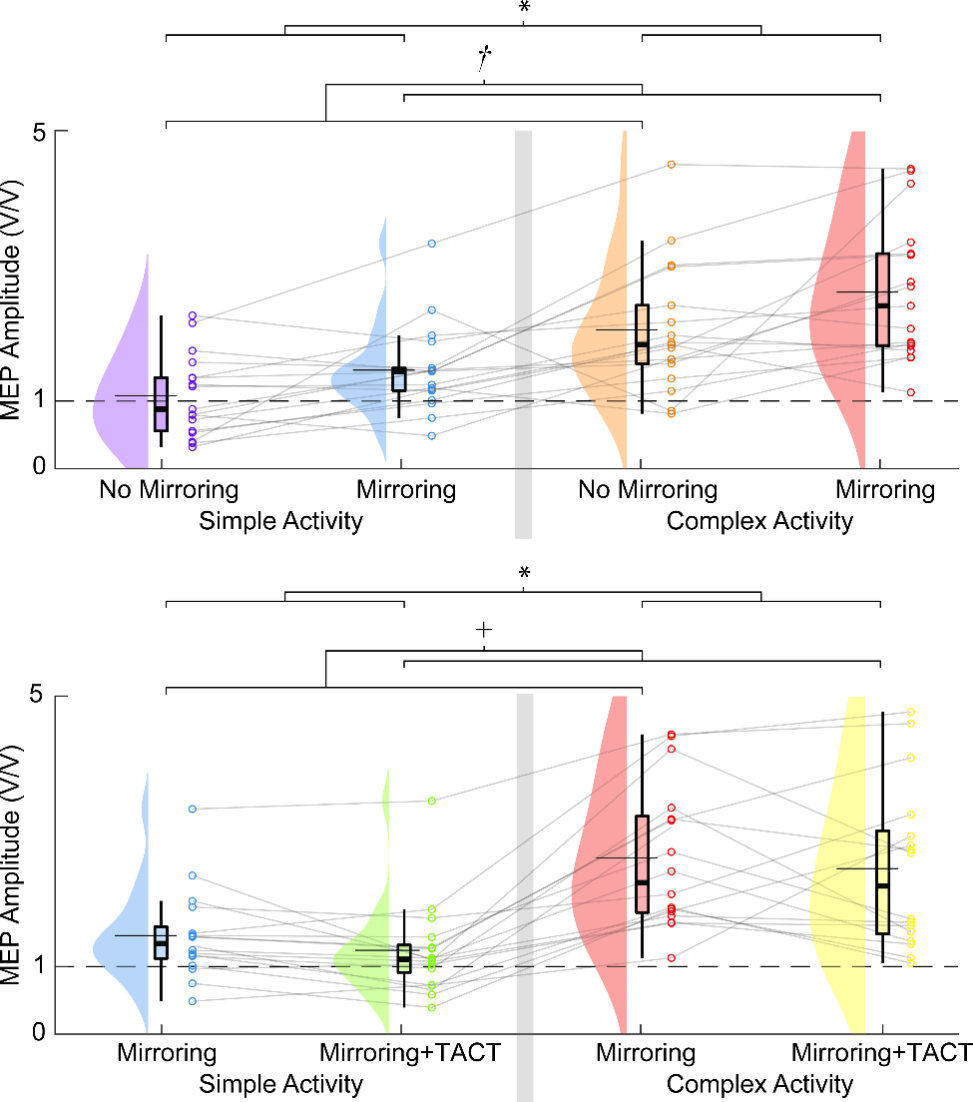
Raincloud plots [76] of our mixed-model analysis comparing the interaction between mirroring and task complexity (Top) and task complexity and visuo-tactile stimulation (Bottom). Here, the thick horizontal line contained by the box-and-whisker indicates the median and the thin horizontal line extending beyond the box-and-whisker indicates the mean. The “*” denotes a significant main effect of task complexity and the “*†*” denotes a significant main effect of mirroring. The significance level was *p* < 0.05

When examining the analysis of mirroring and task complexity, we found significant main effects of mirroring (*F*_*1,48*_ = 14.485, *p* < 0.001) and task complexity (*F*_*1,48*_ = 60.996, *p* < 0.001), but no significant interaction effect (*F*_*1,48*_ = 0.455, *p* = 0.503) (Fig. 4). Post-hoc comparison of the significant main effect of mirroring revealed that normalized MEPs during mirroring were larger than MEPs without mirroring (Δ = 0.323 ± 0.084 V/V ln units). Post-hoc comparison of the significant main effect of task complexity revealed that MEPs were larger during complex activities (Δ = 0.655 ± 0.084 V/V ln units).

When examining the analysis of task complexity and visuo-tactile stimulation, we found significant main effects of task complexity (*F*_*1,47*_ = 86.312, *p* < 0.001) and visuo-tactile stimulation (*F*_*1,47*_ = 4.873, *p* = 0.032), but no significant interaction effect (*F*_*1,47*_ = 0.474, *p* = 0.495, Fig. 4). Post-hoc comparison of the significant main effect of task complexity revealed that MEPs were larger during complex activities (Δ = 0.646 ± 0.07 V/V ln units). Post-hoc comparison of the significant main effect of visuo-tactile stimulation revealed that MEPs were smaller following visuo-tactile stimulation (Δ = 0.154 ± 0.07 V/V ln units). While we found no significant interaction between task complexity and visuo-tactile stimulation, MEPs following visuo-tactile stimulation were notably smaller than MEPs without visuo-tactile stimulation during the simple activity (Δ = 0.201 ± 0.10 V/V ln units, *p* = 0.044) but not during the complex activity (Δ = 0.106 ± 0.1 V/V ln units, *p* = 0.293).

## Discussion

The objective of this study was to examine how VR-enabled modifications to mirror therapy altered corticospinal excitability. We found that corticospinal excitability during unilateral mirroring (i.e., mirroring limb is moving, mirrored limb is immobile) increased relative to no mirroring. We also found that performing a complex, task-oriented activity during mirroring profoundly increased corticospinal excitability, and that a brief period of visuo-tactile stimulation prior to mirroring decreased corticospinal excitability. Our mixed-model analysis, however, found no interaction between these features. These findings reveal the unique ability of virtual reality to easily augment/alter mirror therapy and may have important implications for the use of VR-enabled mirror therapy in a clinical setting.

One interesting finding of our study was that complex, task-oriented activities during mirroring substantially increased corticospinal excitability beyond mirroring itself, suggesting that task-relevant activities may supplement gains from mirror therapy. Our mixed-model analysis, however, revealed no interaction between mirroring and task-relevance, suggesting that the increased excitability from the simple to complex activities in the mirroring condition was similar to the increased excitability from the simple to complex activities when no mirroring illusion is present. While no study has previously examined the interaction between task complexity and mirroring on corticospinal excitability, previous research using functional near-infrared spectroscopy has found that task complexity influences neural activity in the hemisphere contralateral to the mirrored limb during mirror therapy [16, 44, 45]. Therefore, our current study extends these findings by showing that these neural changes result in facilitated corticospinal excitability in the mirroring limb, which could have important implications for mirror therapy in clinical practice [46–48]. Because the observed facilitation of corticospinal excitability with increased task complexity was not primarily mediated by (i.e., did not interact with) mirroring, it is possible that this increase was due to increased ipsilateral corticospinal excitability during motor control tasks. For example, previous studies have shown task-dependent increases in ipsilateral excitability during task-oriented activities involving multiple joints [49, 50] but not during simple activities [51, 52]. Additionally, our findings could also be explained by the attentional demands while stacking the blocks [51, 53–55]. It is important to note that the increase in corticospinal excitability was quite profound (more than 200%) and exceeded many of the priming modalities that are discussed in the literature (e.g., neuromodulatory techniques such as transcranial direct current stimulation [tDCS] or repetitive-TMS) [56, 57]. Thus, complex activities of the less-affected limb prior to more-affected limb therapy could serve as a potential priming technique to induce restoration of limb function after a neurological injury such as stroke. However, it is important to note that these increases in corticospinal excitability were acute, online effects, and it is currently unclear if they would enhance a long-term mirror therapy intervention. As such, further research in this area is necessary to fully understand how VR enabled task-oriented activities can enhance mirror therapy interventions.

We also found that the visuo-tactile stimulation decreased corticospinal excitability during mirroring. Corticospinal excitability has not previously been examined during mirroring following visuo-tactile stimulation, therefore we cannot compare these results with previous, similar studies. However, our findings do align with existing research examining embodiment of fake/prosthetic limbs. These studies mask a participant’s physical limb with a fake/rubber limb, and then provide visuo-tactile stimulation to both limbs (e.g., brushing both limbs with a feather) to create a sense of ownership of the rubber limb (i.e., “rubber-hand illusion”). These studies found that corticospinal excitability was reduced by the rubber hand illusion [58]. The neurological mechanisms leading to the reduced corticospinal excitability following visuo-tactile stimulation are somewhat unclear and may have implications for mirror therapy in clinic. Regarding these mechanisms, it is possible that the reduced excitability could be explained using the same arguments made by researchers who observed reduced excitability following the rubber hand illusion: multisensory feedback increases ownership of the rubber limb (the avatar’s limb in our case), and as result, reduces ownership of the physical limb [58]. Some researchers attribute this reduced ownership to a spatial mismatch between visual feedback of the rubber limb and proprioceptive feedback of the physical limb (i.e., visuo-proprioceptive mismatch), causing the proprioceptive estimate to drift towards the rubber limb [59]. Interestingly, we observed a reduction in excitability with no such spatial mismatch, as the mirroring limb was superimposed directly on top of the physical limb. Furthermore, previous studies have also reported reduced corticospinal excitability following tactile stimulation without an accompanying rubber-hand illusion [60–62]. Therefore, it is possible that the reduced corticospinal excitability is instead mediated by the somatosensory cortex [60], although this modulation may vary (i.e., increase or decrease) with the type of tactile stimulation as alternative tactile stimulations like simulated water flow or mechanical stimulations have been shown to increase excitability [63, 64].

Regardless of their origin, the reduced corticospinal excitability following visuo-tactile stimulation observed in this study does call into question some sentiment in the field of rehabilitation regarding the relative importance of limb agency and ownership to mirror therapy. Specifically, previous studies examining mirror therapy argue that perceptions of both limb agency (i.e., the mirrored limb is controlled by the patient) and ownership (i.e., the mirrored limb is part of the patient’s body) are necessary for mirror therapy [17, 23]. However, our findings suggest that only limb agency enhances corticospinal excitability while limb ownership inhibits it, therefore, visuo-tactile stimulation may inhibit gains in a long-term intervention. This is an important finding as limb ownership becomes increasingly used in other areas of VR-enabled rehabilitation [65, 66] and VR systems become increasingly used in mirror therapy interventions [67–69]. However, it is important to note that even though body ownership may not enhance corticospinal excitability in mirror therapy, it may have utility in other areas of rehabilitation [70, 71]. These results, therefore, highlight important neurological distinctions between body agency and ownership in the context of mirror therapy and outline different use cases of virtual reality in addressing different symptoms of neurological injuries. Future investigations on potential techniques capable of increasing body ownership without inhibiting corticospinal excitability may also be valuable to improving long-term effectiveness of VR-based mirror therapy interventions. Additionally, it is important to note that we did not collect subjective information on the participants’ sense of body ownership in this analysis, so future research comparing excitability changes with subjective feedback could be informative.

The findings in this study suggest several practical applications for virtual reality in the clinic. For instance, we found that VR-enabled mirror therapy increased corticospinal excitability, suggesting that a mirror therapy intervention with VR could be an effective way for clinicians to improve motor outcomes in patients. Because the changes are observed via increased corticospinal excitability, this would suggest that a long-term VR-enabled mirror therapy intervention in clinic could facilitate neuroplastic changes in the ipsilesional corticospinal tract and thus generalize gains to many functional activities. We also found that complex activities with the ipsilateral limb produced a large increase in corticospinal excitability, suggesting that such activities could be an effective approach to improve outcomes of mirror therapy-based interventions in clinic. Additionally, as mentioned previously, we found that this increase was quite profound and observed even without mirroring (even exceeding excitability increases from simple mirroring), which indicates that performing complex activities with the less-impaired arm may be an effective yet accessible priming technique to increase the effectiveness of other interventions in the clinic (similar to the use of neuromodulatory techniques such as tDCS [56]). Interestingly, we found that visuo-tactile stimulation prior to mirroring reduced excitability, suggesting that clinicians should use caution if considering the use of multi-sensory feedback prior to mirror therapy, as outcomes may be inhibited.

We recommend that readers interpret the results of this study with caution while generalizing it to a broader patient population. We note that our findings are from neurologically intact individuals, and there are several factors that may influence the translation to clinical populations like stroke. For instance, all mirroring performed in this study reflected movements across the body with a 1:1 ratio (e.g. a 45° movement of the mirrored limb produced a 45° movement of the mirroring limb) and did not consider input from the nondominant limb. In an intervention with stroke survivors, these characteristics may limit outcomes of a long-term intervention because movements of the mirroring limb in VR will (i) be much larger than the capabilities of the more-impaired limb and (ii) not incorporate movements of the more-impaired limb, thus making the illusion appear less believable. In our previous work, we have presented alternative, VR-enabled alterations to mirror therapy: (i) scalable mirroring to alter movement magnitude, and (ii) shared control to share control of the mirroring limb between the patient’s more- and less-impaired limbs as potential approaches to mitigate this issue [36]. Additionally, participants demonstrated variable behavior between the simple and complex activities, which we believe could be due to ceiling effects or differences in the mental effort between the participants. Therefore, it is possible that a long-term intervention should consider task-difficulty to optimize gains without discouraging patients [72]. Furthermore, stroke survivors with visual field cuts and vestibular impairments may prefer not to use an immersive headset [73, 74], thereby necessitating alternative means of producing the illusion (e.g., a television) that will likely limit movement excursions and therefore could limit benefits of an intervention. Many stroke survivors also demonstrate reduced sensation in the more-impaired limb [75], and therefore visuo-tactile stimulation could have a different effect on corticospinal excitability than in uninjured controls. Finally, it is possible that the observed changes in excitability are partially attributable to a cross-sensory recalibration phase that would reduce after several days or weeks of VR mirror therapy. Therefore, longitudinal studies in clinical population that assess the long-term impact of VR-based mirror therapy and the alterations that VR enables on corticospinal excitability and motor recovery is necessary to fully elucidate the practical clinical application of these findings.

In conclusion, we performed a study to examine how VR-enabled changes (i.e., mirroring, task complexity, and visuo-tactile stimulation) to mirror therapy altered corticospinal excitability. We found that unilateral mirroring increased corticospinal excitability relative to no mirroring, integrating complex activities increased corticospinal excitability during mirroring, and visuo-tactile stimulation prior to mirroring decreased corticospinal excitability. These findings provide insight into the unique ways that virtual reality can enhance or inhibit mirror therapy as well as provide important evidence for the neurological mechanisms underlying the mirroring illusion.

## Data Availability

No datasets were generated or analysed during the current study.
